# Immunoscreening of *Plasmodium falciparum* proteins expressed in a wheat germ cell-free system reveals a novel malaria vaccine candidate

**DOI:** 10.1038/srep46086

**Published:** 2017-04-05

**Authors:** Masayuki Morita, Eizo Takashima, Daisuke Ito, Kazutoyo Miura, Amporn Thongkukiatkul, Ababacar Diouf, Rick M. Fairhurst, Mahamadou Diakite, Carole A. Long, Motomi Torii, Takafumi Tsuboi

**Affiliations:** 1 Division of Malaria Research, Proteo-Science Center, Ehime University, Matsuyama, Ehime, 790-8577, Japan; 2 Laboratory of Malaria and Vector Research, National Institute of Allergy and Infectious Disease, National Institutes of Health, Rockville, Maryland, 20852, USA; 3 Department of Biology, Faculty of Science, Burapha University, Chonburi, 20131, Thailand; 4 Malaria Research and Training Centre, Faculty of Medicine, Pharmacy and Odonto-stomatology, University of Sciences, Techniques, and Technologies of Bamako, Point G, BP 1805, Mali; 5 Division of Molecular Parasitology, Proteo-Science Center, Ehime University, Shitsukawa, Toon, Ehime, 791-0295, Japan

## Abstract

The number of malaria vaccine candidates in preclinical and clinical development is limited. To identify novel blood-stage malaria vaccine candidates, we constructed a library of 1,827*P. falciparum* proteins prepared using the wheat germ cell-free system (WGCFS). Also, a high-throughput AlphaScreen procedure was developed to measure antibody reactivity to the recombinant products. Purified IgGs from residents in malaria endemic areas have shown functional activity against blood-stage parasites as judged by an *in vitro* parasite Growth Inhibition Assay (GIA). Therefore, we evaluated the GIA activity of 51 plasma samples prepared from Malian adults living in a malaria endemic area against the WGCFS library. Using the AlphaScreen-based immunoreactivity measurements, antibody reactivity against 3 proteins was positively associated with GIA activity. Since anti-LSA3-C responses showed the strongest correlation with GIA activity, this protein was investigated further. Anti-LSA3-C-specific antibody purified from Malian adult plasmas showed GIA activity, and expression of LSA3 in blood-stage parasites was confirmed by western blotting. Taken together, we identified LSA3 as a novel blood-stage vaccine candidate, and we propose that this system will be useful for future vaccine candidate discovery.

WHO estimated 214 million cases and 438,000 deaths from malaria in 2015, and *Plasmodium falciparum* was responsible for much of this morbidity and mortality^1^. The emergence of drug-resistant parasites and insecticide-resistant mosquitoes has greatly hampered malaria control and has accelerated development of new approaches to support malaria eradication and elimination efforts^2^,^3^. Moreover, classic studies showing that passive transfer of γ-globulin isolated from adults who lived in a malaria endemic area dramatically reduced parasitemias and alleviated the symptoms in malaria-infected children have pointed to the role of antibodies in protective immune responses^4^. However, the targets of this naturally acquired immunity are not fully understood.

Malaria vaccine candidates under preclinical and clinical development are currently limited (WHO rainbow table: http://www.who.int/vaccine_research/links/Rainbow/en/index.html), and several blood-stage vaccines, which have reached to clinical trials, failed to show efficacy in field studies or in controlled human malaria infection models^5^,^6^. Therefore, more candidates for malaria vaccines for further blood-stage vaccine development are needed. When attempted, the screening for novel candidates has been hampered in part by difficulties in expressing plasmodial proteins in heterologous expression systems. Correct protein conformations are essential to induce protective immunity against malaria^7^, and it is well acknowledged that recombinant plasmodial proteins cannot always elicit functional antibodies due to the lack of proper conformation^8^. There are several preceding studies to detect a broader range of antigen-specific immune responses using protein microarrays^9^,^10^,^11^, and an *Escherichia coli*-based cell-free protein expression system was utilized in those studies to produce the polypeptides. The wheat germ cell-free system (WGCFS) has also been used to express several malaria antigens, and the recombinant proteins produced could elicit *in-vitro* growth inhibitory antibodies in animals as judged by *in vitro* functional assays with *P. falciparum* parasites, such as the growth inhibition assay (GIA)^8^,^12^. The results indicate that the recombinant proteins expressed by WGCFS retain (at least in part) critical functional epitopes. Purified IgGs from residents in malaria endemic areas have shown GIA activities^13^,^14^. To identify novel blood-stage malaria vaccine candidates, in this study 1,827*P. falciparum* proteins were expressed using WGCFS, and 51 purified IgGs were prepared from Malian adults who lived in a malaria endemic area. Antigen-specific antibody reactivity of the IgGs against the 1,827 proteins was evaluated using the AlphaScreen procedure. AlphaScreen is a protein-immobilization free procedure which is likely to maintain protein conformation better than the immobilization methods conventionally used in arrays and which allows high-throughput detection of protein-protein interactions^15^. Using the two data sets, the correlation between antibody responses and GIA activity was evaluated for each protein. We found that the antibody reactivity against three proteins was positively associated with GIA activities. Among the three, anti-C-terminal region of LSA3 (LSA3-C) responses showed the most significant correlation with the GIA activity. Human anti-LSA3-C-specific antibodies purified from Malian adult plasma also showed GIA activity. Previously, LSA3 has been evaluated as a pre-erythrocytic vaccine candidate, and demonstrated a protective effect against *P. falciparum* sporozoite challenge in a chimpanzee^16^ and an *Aotus* monkey model^17^. Moreover, it has been shown that LSA3 genomic sequence is conserved in the isolates from diverse geographical areas compared to other blood-stage vaccine candidate^18^. In the present study, we have shown that LSA3 was expressed in blood-stage parasites judged by western blotting, and immunoelectron microscopy showed that LSA3 was localized to the dense granules of merozoites. Taken together, we identified LSA3 as a novel blood-stage vaccine candidate through this study.

## Results

### Immunoreactivity of the recombinant proteins with Malian adult IgGs

**Figure 1:**
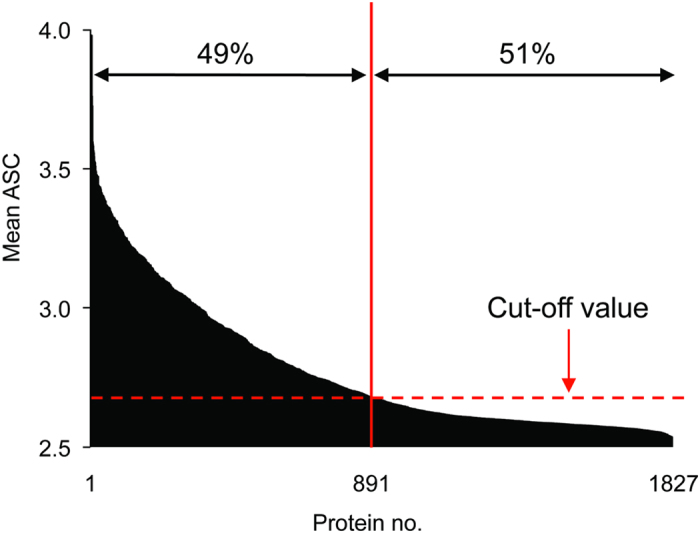
Reactivity of Malian adult IgGs to recombinant proteins produced in the WGCFS. Proteins were sorted by their mean ASC, and the cut-off value was determined as mean plus 3SD of negative control antibodies (dashed line). Approximately 49% of proteins were considered reactive to the Malian IgG samples.

A total of 1,827 recombinant proteins mono-biotinylated at the N-terminus were expressed using WGCFS (Table S1). Antigen-specific IgG responses to these proteins were profiled by an AlphaScreen as described^15^ and shown schematically in Fig. S1. Immunoreactivity of each test IgG against each antigen was determined, and a mean ASC (log-transformed AlphaScreen count) for each antigen was calculated. A total of 891/1,827 (49%) proteins were considered as immunoreactive to the 51 Malian IgG samples tested (Fig. 1), and the immunoreactive antigens were subjected to subsequent analysis.

### Correlation between GIA activity and antibody response

ASC values for individual antigens were fitted to a Gaussian distribution (data not shown). GIA activity of the 51 IgG samples also presented as a Gaussian distribution (Fig. S2). Therefore, we performed a Pearson correlation test for each individual antigen to determine the correlation between ASC and GIA activity. Because the target molecules of GIA are likely to be exposed to the extracellular environment we selected the proteins harboring a transmembrane domain (TM) and/or signal peptide (SP) from the 891 immunoreactive proteins. As a result, out of the 325 selected proteins, the ASC of 3 proteins showed a significant positive correlation with GIA activity (Table 1, Table S2). These positive proteins were LSA3-C, RAMA, and GLURP. No ASC showed a negative correlation with GIA activity as above. Since ASC of LSA3-C most highly correlated with GIA activity, we further characterized LSA3 in blood stage parasite.

### GIA activity of human anti-LSA3-C-specific antibody

**Figure 2:**
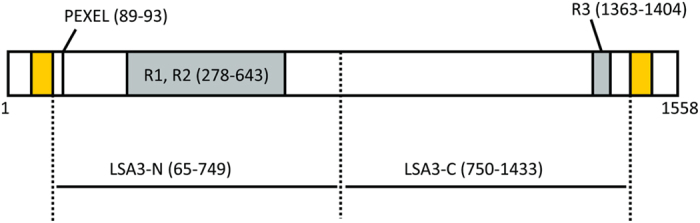
Schematic representation of the *P. falciparum* LSA3 protein. The design of the recombinant proteins expressed; repeat sequence regions (R1, R2, R3), PEXEL motif, and predicted transmembrane domains (in yellow) are shown. The numbers are amino acid positions. The boundaries of the repeat regions were defined by comparison of the primary structure in LSA3 between 3D7 and K1 strain^40^.

**Figure 3:**
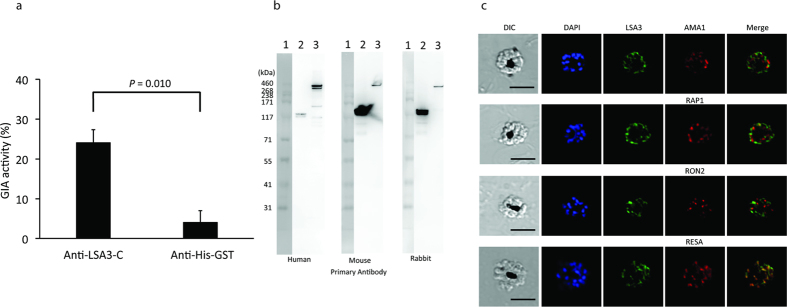
Characterization of LSA3 in blood stage parasites. (**a**) Human anti-LSA3-C-specific IgG has significant GIA activity. Human anti-LSA3-C specific IgG was tested at 0.48 mg/ml. Rabbit anti-His-GST IgG was tested at 20 mg/ml as a negative control. Three independent GIA experiments in triplicate were performed, and the mean and SEM are shown. **(b**) LSA3 was expressed in blood-stage parasites. Western blot was performed with human, mouse or rabbit anti-LSA3-C antibodies. Parasite lysate was obtained from 1 × 10^4^ *P. falciparum* infected red cells at mixed developmental stages. Molecular weight marker (Lane 1), purified 0.5 ng of C-terminal His-tagged recombinant LSA3-C protein (Lane 2) and the blood-stage parasite lysate (Lane 3) were separated by SDS-PAGE under reducing conditions. **(c**) IFA of schizont-stage parasites. Human anti-LSA3-C antibody was used for the IFA. Mouse anti-AMA1, -RAP1, -RON2, or -RESA antibodies were used for counter-staining to determine subcellular localization of microneme, rhoptry body, rhoptry neck or dense granule, respectively. Parasite nuclei were stained with DAPI. Scale bars indicate 5 μm.

ASC of LSA3-C (V_750_ to K_1433_ of LSA3, Library ID:1705, PF3D7_0220000, Fig. 2) highly correlated with GIA data using Malian adult total IgGs (Table 1), suggesting that anti-LSA3-C antibody has functional activity against blood-stage parasites. However, it had earlier been thought that the LSA3 antigen was expressed only in pre-erythrocytic-stage parasites^16^. Therefore, we next determined the functional activity of human anti-LSA3-C-specific antibodies affinity purified from Malian adult total IgGs. Since the volume of antigen-specific IgG was limited, the human anti-LSA3-C-specific IgG was tested at a single concentration (0.48 mg/ml, which was the highest concentration that could be tested). The antigen-specific IgG exhibited 24% inhibition (Fig. 3a), which was significantly higher than that of the negative control (Student’s t-test; p = 0.010).

### Characterization of LSA3 in blood stage parasites

We then characterized LSA3 in the blood-stage parasite. Expression of LSA3 in the blood-stage parasites was first tested by western blotting with trophozoite/schizont-rich 3D7 parasite lysate using the purified human anti-LSA3-C antibody (Fig. 3b). Although the predicted molecular weight of LSA3 is 175 kDa, these antibodies specifically visualized two protein bands of approximately 300 kDa. Consistent with the unexpectedly high molecular weight of LSA3 observed in the western blot, recombinant LSA3-C whose size is predicted to be 80 kDa, migrates at approximately 120 kDa in SDS-PAGE (Fig. 3b, Fig. S3). When western blotting analysis was performed with mouse and rabbit antibodies, which were raised against purified N-terminal GST tagged LSA3-C protein, the same high molecular weight bands were observed (Fig. 3b). Since the sizes of bands determined by the SDS-PAGE were larger than expected, to confirm the specificity of anti-LSA3-C antibody, we performed two experiments. A rabbit was immunized with N-terminal LSA3 protein (LSA3-N, Fig. 2), and western blotting analysis with the anti-LSA3-N antibody specifically visualized protein bands of approximately 300 kDa (Fig. S4) as consistent with the result of anti-LSA3-C antibody. In the second experiment, immunoprecipitation was performed with rabbit anti-LSA3-C antibody using rabbit anti-His-GST antibody as a negative control. The anti-LSA3-C antibody pulled down a protein band which migrated around 300 kDa. Mass spectrometry (MS) analysis of the band detected 7 specific peptides which were only recognized in anti-LSA3-C sample, and 6 of the peptides were matched with LSA3 sequence (Fig. S5). Taken together, the data suggest native LSA3 protein, which is expressed in blood-stage parasites, does not migrate according to the predicted molecular weight.

**Figure 4:**
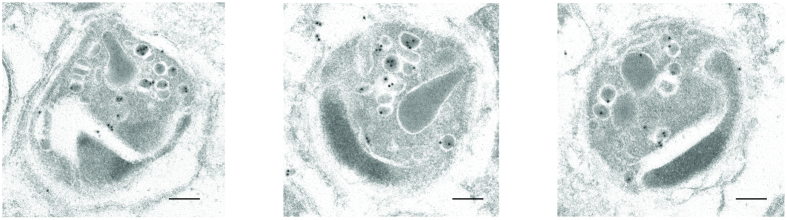
Immunoelectron microscopy observations of LSA3 in merozoites of schizont-infected erythrocytes. Rabbit anti-LSA3-C antibody was used for primary antibody. The gold particles were detected specifically at dense granules. Scale bars indicate 200 nm.

We further examined the subcellular localization of LSA3 by IFA. As shown in Fig. 3c, LSA3 in schizont-stage parasites co-localized with RESA, suggesting that LSA3 localized to dense granules of merozoites. To confirm this observation, immunoelectron microscopy using rabbit anti-LSA3-C antibody was conducted. As shown in Fig. 4, LSA3 localized to the dense granules of merozoites formed in schizont parasites. IFA results using rabbit anti-LSA3-C antibody were consistent with those using human antibody (Fig. S6).

## Discussion

We generated a large *P. falciparum* protein library encompassing much of the asexual parasite proteome for the first time using the WGCFS expression system, and this library was screened with antibodies from Malian adults having naturally acquired antibody responses as a result of repeated malaria infections. Antigen-specific antibody responses against 3 proteins, out of 325 immunoreactive ones with TM and/or SP tested, showed significant correlations with the functional activity measured by GIA. One of the key aspects of this study is that we quantified antigen-specific antibody responses using the AlphaScreen that is an immobilization-free method to detect immunoreactions. We have shown for the first time that human antibody against LSA3 (more specifically LSA3-C), which was selected from this study, showed functional activity. The positive GIA result indicates the screening strategy shown in this study is valuable for a future novel blood-stage malaria vaccine candidate discovery.

Although *P. falciparum* genome information has been available for more than a decade, parasite antigens targeted by anti-malaria immunity in humans remains largely unknown. This bottleneck has partially been attributed to difficulties in expression of the plasmodial proteins. Several studies have aimed to profile host antibodies using protein microarrays containing recombinant *P. falciparum* proteins synthesized by *E. coli*-based cell-free protein expression systems^10^,^19^. However, a major drawback of these high-throughput protein microarray based approaches may be the conformation of expressed proteins using this system. To the best of our knowledge, none of the malaria recombinant proteins expressed by the *E. coli*-based cell-free protein expression systems has been shown to induce functional antibodies without codon-optimization and/or a refolding process. In contrast, WGCFS is a eukaryotic plant-based recombinant protein expression system that may offer a better alternative. The system can synthesize recombinant proteins that retain critical functional epitopes, at least in part, as evidenced by the fact that antibodies elicited against the recombinant proteins showed functional activities with live parasites^20^,^21^. WGCFS therefore offers a unique and reliable platform for identification of plasmodial proteins that could be important targets of host humoral immune responses. The AlphaScreen-based antibody-antigen reactions take place in homogenous solution rather than in a solid phase and thus this is a sensitive platform for detection of protein-tertiary-structure-dependent antibody immunoreactions^22^. Here we applied the WGCFS and the AlphaScreen to comprehensively analyze immunoreactions of IgGs obtained from malaria-exposed individuals in Mali. We detected immunoreactivity in approximately half of the proteins (891/1,827), which is higher than the reactivity reported in previous studies (~20%)^10^,^19^. While a further head-to-head study is required to compare the two systems directly, the higher immunoreactivity seen in this study might be partially explained by the maintenance of better conformation of proteins in the WGCFS/AlphaScreen system.

To correlate this immunoreactivity with a functional anti-parasite response, we compared these results with the GIA activity data; in this analysis the anti-LSA3-C antibody response showed the most significant correlation with GIA activity (Table 1). In the present study, we synthesized 2 truncated recombinant proteins of LSA3, because a full-length LSA3 protein is too large for WGCFS expression, in terms of the molecular weight (Fig. 2). Although antigen polymorphism is a major obstacle in development of vaccine against blood-stage parasite^23^, according to the sequences available in PlasmoDB (http://plasmodb.org/), LSA3-C region is conserved compared with other blood-stage vaccine candidates (dNS/dS = 3.3); in contrast the LSA3-N region is relatively polymorphic (dNS/dS = 6.4). A previous study also showed that non-repeat regions of LSA3 gene sequence are highly conserved in the isolates from diverse geographical areas^18^.

Since GIA activity of anti-LSA3 antibody had not been reported in any species, we affinity purified human anti-LSA3-C-specific antibody and confirmed that the antibody could show GIA activity. The fact that human anti-LSA3-C specific antibody showed GIA activity suggested that the LSA3-C recombinant protein expressed by WGCFS conserved, at least in part, native conformation of the protein in parasites. However, the test concentration in GIA was at least 10-fold higher than seen in human plasmas (data not shown). Therefore, further study is required to determine contribution of each antigen-specific antibody to the GIA activity observed with total IgGs. For unknown reasons, the rabbit anti-LSA3-C antibody did not show any GIA activity (data not shown). Since we could purify functional human antibody with the LSA3-C, the LSA3-C contains at least one functional epitope which is the target of inhibitory human antibody. In the rabbit study, the animal was immunized with truncated LSA3 protein (LSA3-C), which could express different epitopes which were not seen in the (native) full-length LSA3. Thus it could be possible that the immunoreactivity against such artificial epitopes on LSA3-C masked the reactivity against the functional epitope(s) in rabbit when immunized with LSA3-C. Alternatively, it might be also possible that the epitope recognition is not necessarily the same in rabbits and humans for some antigens.

In this study, we have shown that LSA3 is expressed in blood-stage parasites by western blotting analysis, consistent with previous MS/MS analysis that detected LSA3 in ring, trophozoite, schizont, and merozoite stage parasites^24^,^25^. The molecular weight estimated from migration in the gel was higher than predicted from amino acid sequence (approximately 175 kDa). In Fig. S3, the recombinant LSA3-C expressed by WGCFS migrated at approximately 120 kDa in SDS-PAGE, which was ~40 kDa heavier than expected (approximately 80 kDa). Because WGCFS is free from glycosylation systems^26^, the observed slow migration of recombinant LSA3-C protein is likely due to the physiological properties of the LSA3 protein itself. This might explain, at least in part, the reasons why native LSA3 did not migrate at the expected molecular weight. On the other hand, LSA3 might be transcriptionally or post-translationally modified at sporozoite-stage, because the previous western blotting with sporozoite lysate detected a ~175 kDa protein band of LSA3^16^.

Immunoelectron microscopy clearly showed that LSA3 is localized in dense granules (Fig. 4). It has been reported that dense granules secrete their contents into the forming parasitophorous vacuole (PV) during merozoite invasion^27^. Consistent with other dense granule proteins^28^, LSA3 is located at the PV of ring-stage parasites (Fig. S7). This is also in line with a previous study showing that LSA3 is localized to the PV of 5- and 6-day-old liver-stage schizonts in the chimpanzee’s liver cells^16^. Therefore, LSA3 could have a common function during parasite development in both hepatocytes and erythrocytes.

The results reported here are from a limited number of field IgG samples, thus it would be worthwhile to validate the screening results using a larger number of samples and samples from widely different malaria endemic area. A previous cohort study using a *P. falciparum* protein microarray by other investigators has suggested that higher titers against LSA3 might contribute to the prevention of symptomatic malaria infections during 12 weeks of follow-up after drug treatment^29^. However, another study using a different *P. falciparum* protein microarray showed that the levels of anti-LSA3 antibodies did not correlate with subsequent malaria risk during a period of seasonal transmission in 8–10 year old Malian children^10^. Although GIA has been utilized to identify novel vaccine candidates and prioritize vaccine formulations, there are also reports showing no correlation between GIA activity and clinical protection in several epidemiological studies^30^. Therefore, further studies are anticipated using the WGCFS/AlphaScreen system established in this study to identify the targets of naturally acquired protective antibody responses. Such studies will include human samples obtained from carefully designed cohort studies and samples assessed by other functional assays. These future studies may identify additional novel malaria vaccine candidates and enhance our understanding of clinical immunity to asexual stages of malaria infections.

## Materials and Methods

### Production of parasite proteins

We created a large *P. falciparum* protein library comprised of 1,827 polypeptides derived from 1,565 genes. We first amplified 554 genes by PCR using LATaq PCR kit (Takara Bio, Kusatsu, Japan) and cloned the products into the pCR2.1-TOPO vector (Invitrogen, Carlsbad, CA). Genes encoding an additional 148 proteins were amplified by PCR and cloned into the pEU vector (CellFree Sciences, Matsuyama, Japan). The library also contained an additional 1,125 target proteins derived from full-length cDNA library clones raised from trophozoite-schizont rich 3D7 *P. falciparum* cultures. The recombinant proteins were expressed without codon optimization using the WGCFS as previously described^31^. WGCFS synthesis of the *P. falciparum* protein library was based on the previously described bilayer diffusion system^32^. For biotinylation of proteins, 500 nM D-biotin (Nacalai Tesque, Kyoto, Japan) was added to both the translation and substrate layers. Crude wheat germ cell-free expressed BirA (1 μl) was added to the translation layer. *In vitro* transcription and cell-free protein synthesis for the *P. falciparum* protein library were carried out using the GenDecoder 1000 robotic synthesizer (CellFree Sciences) as previously described^33^,^34^. Expression of the proteins was confirmed by western blotting using FITC- or HRP-conjugated streptavidin (ThermoFisher Scientific, Waltham, MA, Code # SA10002 or #21130, respectively).

### Detection and quantification of *P. falciparum* antigen-specific IgG by AlphaScreen

*P. falciparum* antigen-specific antibodies were quantified by an AlphaScreen as previously reported^15^ with slight modifications. The protocol was automated by use of the JANUS Automated Workstation (PerkinElmer Life and Analytical Science, Boston, MA). Reactions were carried out in 25 μl of reaction volume per well in 384-well OptiPlate microtiter plates (PerkinElmer). First, 0.1 μl of the translation mixture containing a recombinant *P. falciparum* biotinylated protein was diluted 50-fold (5 μl), mixed with 10 μl of 10 μg/ml Malian adult IgG in reaction buffer (100 mM Tris-HCL [pH 8.0], 0.01% [v/v] Tween-20 and 0.1% [w/v] bovine serum albumin), and incubated for 30 min at 26 °C to form an antigen-antibody complex. Subsequently, a 10 μl suspension of streptavidin-coated donor-beads and acceptor-beads (PerkinElmer) conjugated with protein G (Thermo Scientific) in the reaction buffer was added to a final concentration of 12 μg/ml of both beads. The mixture was incubated at 26 °C for 1 h in the dark to allow the donor and acceptor-beads to optimally bind to biotin and human IgG, respectively. Upon illumination of this complex, a luminescence signal at 620 nm was detected by the EnVision plate reader (PerkinElmer) and the result was expressed as AlphaScreen counts. The performance of the AlphaScreen was validated with 200 pM of biotinylated-IgG to be Z-factor with more than 0.5. A translation mixture expressing AMA1 incubated with a plasma sample taken from a Thai malaria-immune individual was used as a positive control in each plate. A translation mixture of WGCFS without template mRNA incubated with a plasma sample taken from a Thai malaria naïve individual was used as a negative control in each plate. Reading the plates was conducted in a randomized manner to avoid biases.

### Production of mouse and rabbit antisera

As previously described^35^, we generated rabbit polyclonal antisera against LSA3-C (V_750_ to K_1433_ of the 3D7 sequence; PF3D7_0220000) and mouse antisera against AMA1 (Q_25_ to K_546_; PF3D7_1133400), RAP1 (M_1_ to D_782_; PF3D7_1410400), and RON2 (K_84_ to Q_968_; PF3D7_1452000). All antigens were synthesized by WGCFS and purified by GST or hexa-histidine (His) protein-tag fused with the N-terminus of the recombinant proteins. Mouse monoclonal antibody against RESA (PF3D7_0102200) was a kind gift from Robin F. Anders^36^.

### Human plasma collection and IgG preparations

A cohort study was conducted between 2008 and 2011 in three villages of Kenieroba, Fourda and Bozokin in southern Mali. Details of the main cohort study are described elsewhere^37^, and the study is registered with ClinicalTrials.gov, number NCT00669084. As a part of the study, plasma samples were collected from 51 healthy adults in October, 2008 (middle of the malaria transmission season). Detailed methods for preparation of total IgG and antigen-specific IgG from Malian plasma samples have been described previously^38^. In brief, total IgG purification was performed using a Protein G column for each plasma sample. For the anti-LSA3-C-specific IgG purification, 4 adult plasma samples with higher anti-LSA3-C titers were pooled first, and the total IgG was applied to a NHS-activated Sepharose 4 Fast Flow column coupled with WGCFS synthesized C-terminal His-tagged LSA3-C protein according to the manufacturer’s instructions.

### Western blot analysis

Western blot was conducted with anti-LSA3-C antibodies as previously described^8^. Purified schizont-rich parasite pellets from *P. falciparum* 3D7 strain were directly lysed in reducing SDS-PAGE sample buffer. The lysate was boiled at 95 °C for 5 min, centrifuged at 10,000 × g for 10 min at 4 °C, supernatants were collected, and resolved by electrophoresis on a 4–12% Bolt Bis-Tris Plus gel (ThermoFisher Scientific) with MOPS buffer. Following electroblotting, the membrane was incubated with primary antibodies diluted at the following concentrations: human anti LSA3-C antibody, 0.173 μg/ml; mouse anti-LSA3-C antibody, 1:300; rabbit anti-LSA3-C antibody, 1:1,000. After washing, it was incubated with secondary antibodies diluted at the following concentrations: polyclonal rabbit anti-human IgG/HRP (Dako, Glostrup, Denmark, Code # P0214), 1:20,000; ECL peroxidase labeled anti-mouse antibody (GE Healthcare, Chicago, IL, Code # NA931), 1:10000; ECL peroxidase labeled anti-rabbit antibody (GE Healthcare, Code # NA934), 1:10,000.

### Indirect immunofluorescence assay (IFA)

IFA was conducted as previously described^35^. The samples were stained with primary antibodies diluted at the following concentrations in blocking solution at 37 °C for 1 h: human anti-LSA3-C antibody, 0.173 μg/ml; rabbit anti-LSA3-C antibody, 1:500; mouse anti-AMA1 antibody, 1:100; mouse anti-RAP1 antibody, 1:1,000; mouse anti-RON2 antibody, 1:100; mouse anti-RESA monoclonal antibody, 1:100. Secondary antibodies, Alexa Fluor 488-conjugated goat anti-human IgG (Invitrogen, Code # A11013), Alexa Fluor 488-conjugated goat anti-rabbit IgG (Invitrogen, Code # A11034) and Alexa Fluor 568-conjugated goat anti-mouse IgG (Invitrogen, Code #A11031), were used at a 1:500 dilution in blocking solution at 37 °C for 30 min. DAPI (4’, 6-diamidino-2-phenylindole) (Invitrogen, Code #D1306) at 2 μg/ml was also added to the secondary-antibody solution to stain the nuclei. Slides were mounted in ProLong Gold Antifade reagent (Invitrogen, Code #P36934) and viewed under a 63 × oil immersion lens. High-resolution image capture and processing were performed with a confocal scanning laser microscope (LSM710; Carl Zeiss MicroImaging, Thornwood, NY). Images were processed in Adobe Photoshop (Adobe Systems Inc., San Jose, CA).

### Immunoelectron microscopy

Parasites were fixed and embedded in LR White resin (Polysciences, Warrington, PA)^39^ and ultrathin sections were immunostained^8^ as previously described. Rabbit anti-LSA3-C antibody was used at a 1:200 dilution. Samples were examined with a transmission electron microscope (JEM-1230; JEOL, Tokyo, Japan).

### *P. falciparum* growth inhibition assay (GIA)

The GIA against 3D7 strain with 10 mg/ml total human IgGs was performed at the National Institute of Allergy and Infectious Diseases (NIAID) as described previously^38^. The GIA with human anti-LSA3-C-specific IgG was performed at Ehime University as described elsewhere^8^. Both GIAs were one-cycle assays, and parasite growth was determined by a biochemical assay at NIAID^38^, and by counting SYBR Green I positive cells with flow cytometry at Ehime University^8^.

### Ethical approval

The Mali cohort study was reviewed and approved by the Institutional Review Boards (IRB) of the NIAID, National Institutes of Health (NIH) and by the Ethics Committee of the Faculty of Medicine, Pharmacy and Odontostomatology, University of Bamako (Protocol no. 08-I-N120, ClinicalTrials.gov identifier no. NCT00669084). The study to obtain serum samples from Thailand was approved by the Ethics Committee of the Thai Ministry of Public Health and the IRB of the Walter Reed Army Institute of Research (WRAIR 802). Written informed consents were obtained from all adult volunteers. The study was conducted in accordance with approved protocols and regulations. All animal experimental protocols were approved by the Institutional Animal Care and Use Committee of Ehime University (su-14-6), and the experiments were conducted according to the Ethical Guidelines for Animal Experiments of Ehime University.

### Statistical analysis

Raw AlphaScreen counts were converted to logarithmic values, and subsequent analyses were performed with the log-transformed values (ASC). The protein whose mean ASC of the 51 test IgG samples was below the cut-off value (mean plus 3 standard deviations of the negative control) was considered as a non-reactive protein and was excluded from further analysis. For the reactive proteins, a Kolmogorov-Smirnov test was used for testing normality of the distribution, and a Pearson correlation test was used to analyze the correlation between GIA activity and ASC for each antigen. Benjamini-Hochberg’s correction was utilized for multiple comparisons. The R software (version 3.1.2; R Foundation for Statistical Computing) was used for statistical analysis and *p* < 0.05 was considered significant.

## Additional Information

**How to cite this article:** Morita, M. *et al*. Immunoscreening of *Plasmodium falciparum* proteins expressed in a wheat germ cell-free system reveals a novel malaria vaccine candidate. *Sci. Rep.* **7**, 46086; doi: 10.1038/srep46086 (2017).

**Publisher's note:** Springer Nature remains neutral with regard to jurisdictional claims in published maps and institutional affiliations.

## Supplementary Material

Supplementary Information

Supplementary Dataset

## Figures and Tables

**Table 1:** The proteins with the positive significant correlations between antibody responses and GIA activity.

Library ID	Gene ID	Gene name	Expressed region	ASC	r	Unadjusted p-value	Adjusted p-value
			(aa)	Mean			
1705	PF3D7_0220000	Liver stage antigen 3C (LSA3-C)	750–1433	2.84	0.571	1.18 × 10^−5^	0.004
1303	PF3D7_0707300	Rhoptry-associated membrane antigen (RAMA)	18–786	3.13	0.475	4.2 × 10^−4^	0.046
829	PF3D7_1035300	Glutamate-rich protein (GLURP)	34–459	3.42	0.486	2.9 × 10^−4^	0.046
