# Supplementary material for: Immunoscreening of Plasmodium falciparum proteins expressed in a wheat germ cell-free system reveals a novel malaria vaccine candidate
Source: Sci Rep. 2017 Apr 5;7:46086. doi: 10.1038/srep46086 (PMC5380959; doi:10.1038/srep46086)

Supplementary Information

Immunoscreening of *Plasmodium falciparum* proteins expressed in a wheat germ cell-free system reveals a novel malaria vaccine candidate

Masayuki Morita[1], Eizo Takashima[1]*, Daisuke Ito[1], Kazutoyo Miura[2], Amporn Thongkukiatkul[3], Ababacar Diouf[2], Rick M. Fairhurst[2], Mahamadou Diakite[4], Carole A. Long[2], Motomi Torii[5], and Takafumi Tsuboi[1]

[1]Division of Malaria Research, Proteo-Science Center, Ehime University, Matsuyama, Ehime, 790-8577, Japan.

[2]Laboratory of Malaria and Vector Research, National Institute of Allergy and Infectious Disease, National Institutes of Health, Rockville, Maryland, 20852, USA.

[3]Department of Biology, Faculty of Science, Burapha University, Chonburi, 20131, Thailand.

[4]Malaria Research and Training Centre, Faculty of Medicine, Pharmacy and Odonto-stomatology, University of Sciences, Techniques, and Technologies of Bamako, Point G, BP 1805, Mali.

[5]Division of Molecular Parasitology, Proteo-Science Center, Ehime University, Shitsukawa, Toon, Ehime, 791-0295, Japan.

*Corresponding Author, Email:takashima.eizo.mz@ehime-u.ac.jp

Materials and methods for supplementary information

Synthesis of recombinant LSA3-N protein and production of rabbit antiserum

As previously described [1], we generated rabbit polyclonal antiserum against LSA3-N (N_{65} to T_{749} of the 3D7 sequence; PF3D7_0220000). The antigen was synthesized by WGCFS and purified by GST tag fused with the N-terminus of the recombinant protein. For control of western blotting, GST-tag was removed with TEV-protease digestion at TEV protease site located between GST-tag and LSA3-N.

MS analysis

Immunoprecipitation was carried out as previously described [2]. Briefly, proteins were extracted from late schizont parasite pellets in TNE buffer (50 mM Tris-HCl, 0.2 M NaCl, 5 mM EDTA) with 0.5 % Nonidet P-40 containing cOmplete™ protease inhibitor (Roche). Seventy five microliter samples of supernatants were pre-incubated at 4°C for 1 h with 36 μl of 50% protein G-conjugated beads (protein G-Sepharose 4 Fast Flow; GE Healthcare) in TNE buffer. Aliquots of recovered supernatants were incubated with 3 μl of rabbit anti-LSA3-C serum or rabbit anti-GST serum at 4°C for 2 h, and then 18 μl of a 50% protein G-conjugated bead suspension was added. After incubation for 2 h at 4°C, the beads were washed three times with TNE buffer. Finally, proteins were extracted from the protein G-conjugated beads by incubation with 1× SDS-PAGE reducing loading buffer at 95°C for 5 min, and subjected to SDS-PAGE. Protein bands were excised from gels and subjected to in-gel digestion with sequence-grade modified trypsin (Promega), as previously described [3]. Tryptic digests were reconstituted with 0.2 % (v/v) trifluoroacetic acid for mass spectrometric analysis. Peptide mass fingerprint (PMF) analysis was performed using a MALDI-TOF/TOF mass spectrometer (Shimadzu AXIMA-TOF2). MS spectrum data were submitted to the MS-Fit (http://prospector.ucsf.edu) in order to identify peptides derived from LSA3. The conditions of the search were as follows; Database searched, amino-acid sequence of LSA3 (PF3D7_0220000); Max number of missed cleavages, 1; Constant

modification, Propionamide (C); Considered mofidications, Peptide N-terminal Gln to pyroGlu, Oxidation of Met, and Protein N-terminus acetylated; MOWSE P factor, 0.4.

Table S1 (Supplementary dataset file)

Information on 1,827 proteins in the library expressed by WGCFS. Cloning method: PCR indicates that the PCR product was cloned into the TA-cloning vector. cDNA designates a clone from a full-length cDNA library. Restriction enzyme represents targets insert cloned into the expression vector for WGCFS.

Table S2 (Supplementary dataset file)

Result of the statistical analysis of the immunoreactive 325 proteins with TM and/or SP. Cloning method: PCR indicates the PCR product was cloned into TA-cloning vector. cDNA designates a clone from full-length cDNA library. Restriction enzyme represents target insert cloned into the expression vector for WGCFS. r represents the Pearson correlation coefficient. CI lower and CI upper show lower and upper boundaries of confidence intervals, respectively. Adjusted P-value was calculated by Benjamini-Hochberg's correction for 325 multiple comparisons.

Figure legends

Figure S1

Schematic presentation of AlphaScreen-based measurement of antigen-antibody response. Human IgG is incubated with mono-biotinylated recombinant plasmodial protein. Following the incubation, the streptavidin conjugated donor beads and protein G conjugated acceptor beads are added to the mixture. When the immune-complex is formed, the excitation of the donor beads causes the release of singlet oxygen molecules that trigger the light emission at 520-620 nm from the acceptor beads.

Figure S2

Distribution of GIA activity of the Malian adult IgG against 3D7 strain.

GIA activities against *P. falciparum* 3D7 strain parasites were measured as described in Materials and Methods using Malian adult IgGs (n=51).

Figure S3

Purified C-terminal His-tagged recombinant LSA3-C protein visualized by Coomassie brilliant blue staining following SDS-PAGE.

Figure S4

Western blot analysis with anti-LSA3-N and C antibodies. Western blot was performed with (a) rabbit anti-LSA3-N serum (1:1000) and (b) rabbit LSA3-C rabbit IgG (0.43 μg/ml). Parasite lysate was obtained from *P. falciparum* infected red cells at mixed developmental stages. LSA3-N (0.25 ng) at the lane 1, LSA3-C (0.25 ng) at the lane 2, and blood-stage parasite lysate derived from 2×10^5 cells at the lane 3 were separated by SDS-PAGE under reducing conditions.

Figure S5

MS analysis of the protein band immunoprecipitated with anti-LSA3-C antibody. **a)** Molecular weight marker (Lane 1), immunoprecipitated sample using rabbit anti His-GST serum (Lane 2, negative control), and rabbit anti LSA3-C serum (Lane 3) were separated by SDS-PAGE under reducing conditions. The gel was stained by CBB. Protein bands indicated by arrow heads were excised for MS analysis. PMFs derived from the 2 samples were compared, then peptides specifically detected from the lane 3 were subjected to database search (jPOSTrepo ID # JPST000238). **b)** Amino acid sequence of LSA3. Out of 7 peptides specifically detected at the sample from the lane 3, 6 peptides were matched with LSA3 sequence. PMF matched peptides were indicated in red font.

Figure S6

Immunofluorescence analysis of schizont parasites using rabbit anti-LSA3-C antibody. Rabbit anti-LSA3-C antibody was utilized for the IFA. Mouse anti-AMA1, -RAP1, -RON2, or -RESA antibodies were used for counter-staining to determine subcellular localization of microneme, rhoptry body, rhoptry neck or dense granules, respectively. Parasite nuclei were stained by DAPI. Scale bars indicate 5 μm.

Figure S7

Immunofluorescence analysis of ring stage parasites using rabbit anti-LSA3-C antibody. Rabbit anti-LSA3-C antibody was used for the IFA. Mouse anti-RAP1 antibody was used for counter-staining to determine subcellular localization of the parasitophorous vacuole (PV). Parasite nuclei were stained by DAPI. Scale bar indicates 5 μm.

Reference

1 Ito, D. *et al*. RALP1 is a rhoptry neck erythrocyte-binding protein of *Plasmodium falciparum* merozoites and a potential blood-stage vaccine candidate antigen. *Infect Immun* **81**, 4290-4298, doi:10.1128/IAI.00690-13 (2013).

2 Ito, D. *et al*. Plasmodial ortholog of *Toxoplasma gondii* rhoptry neck protein 3 is localized to the rhoptry body. *Parasitol Int* **60**, 132-138 (2011).

3 Ohnuki, H. *et al*. BAZF, a novel component of cullin3-based E3 ligase complex, mediates VEGFR and Notch cross-signaling in angiogenesis. *Blood* **119**, 2688-2698 (2012).

Figure S1

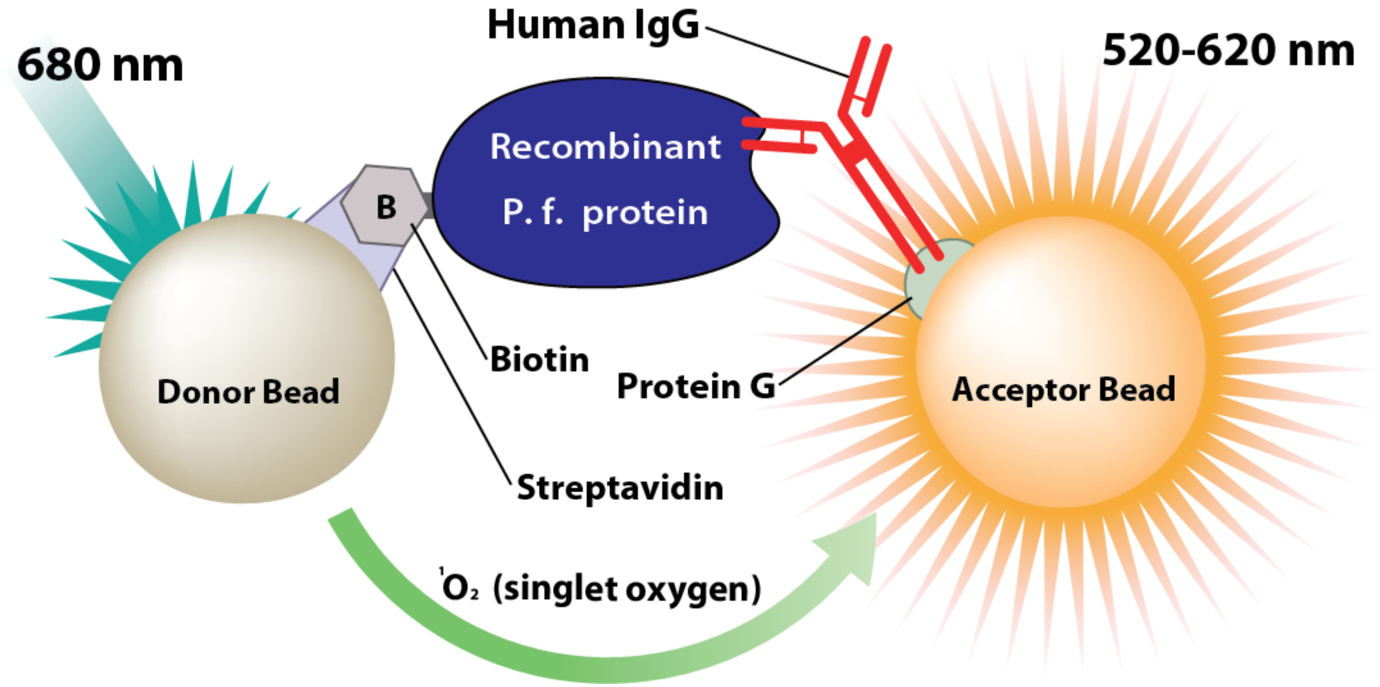

680 nm
Human IgG
520-620 nm
Recombinant
P. f. protein
B
Biotin
Protein G
Donor Bead
Acceptor Bead
Streptavidin
¹O₂ (singlet oxygen)

Figure S2

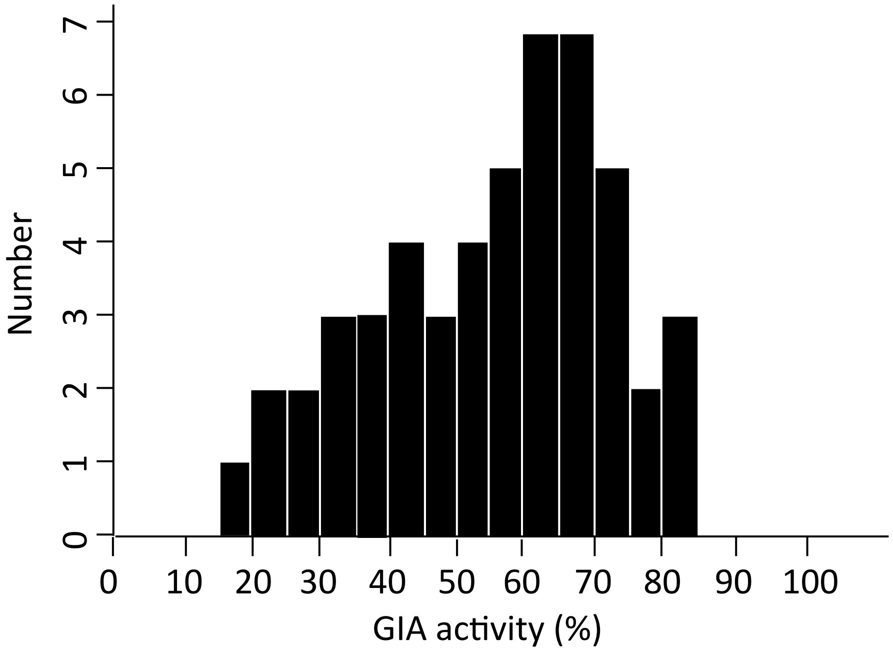

Figure S3

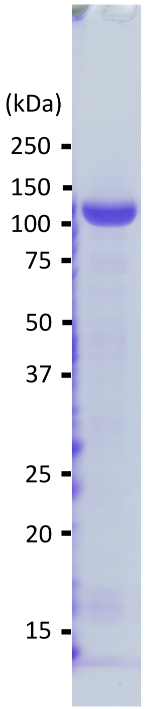

Figure S4

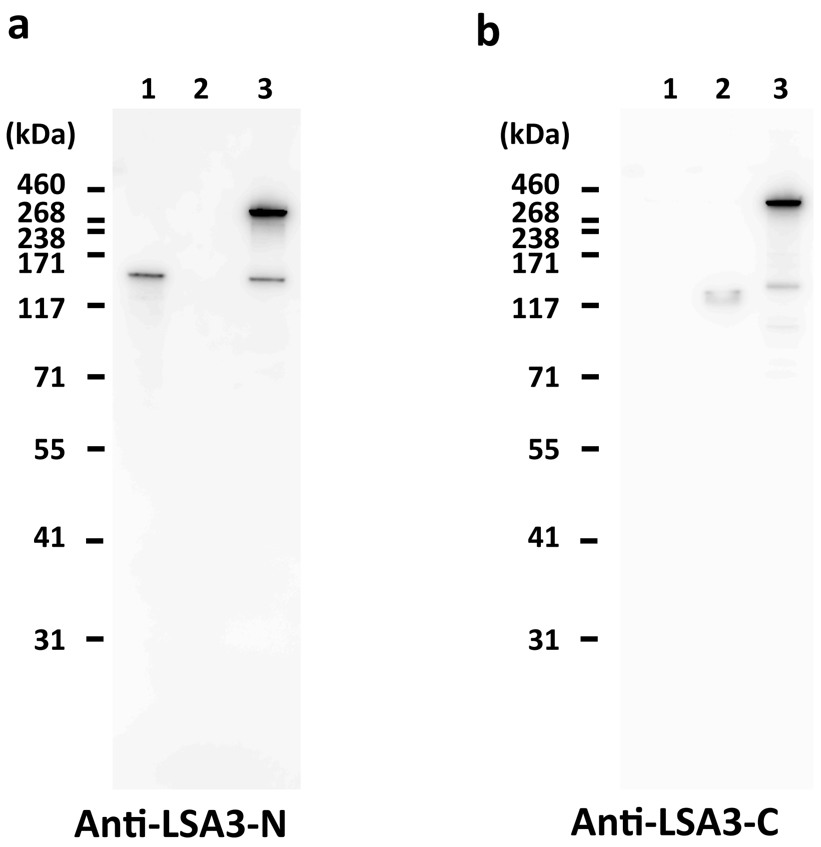
a
1 2 3
(kDa)
460
268
238
171
117
71
55
41
31
Anti-LSA3-N
b
1 2 3
(kDa)
460
268
238
171
117
71
55
41
31
Anti-LSA3-C

Figure S5 a

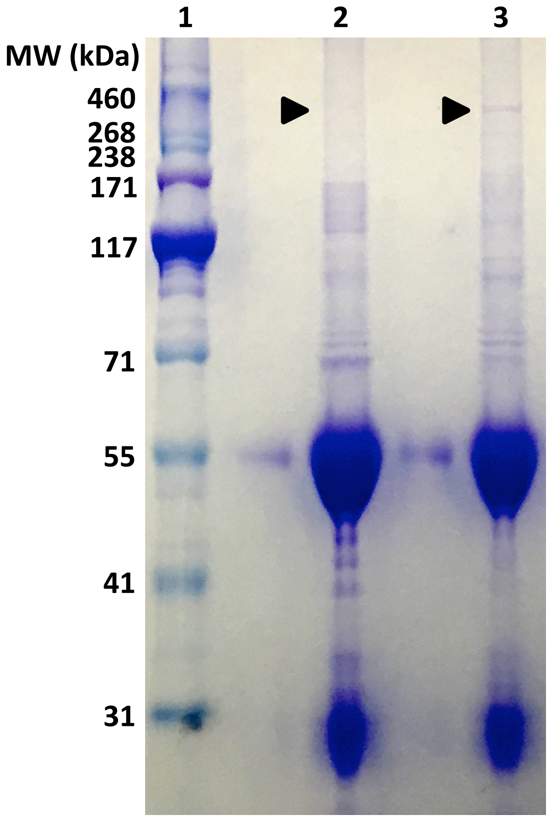

Fig. S5 b

MTNSNYKSNNKTYNENNNEQITTIFNRTNMNPIKKCHMREKINKYFFLIKILTCTILIWAVQYANNSDINKSWKKNTYVDKKLNKLFNRSLGESQVNGELASEEVKEKILDLLEEGNTLTE
SVDDNKNLEEAEDIKENILLSNIEEPKENIIDNLLNNIGQNSEKQESVSENVQVSDELFNELLNSVDVNGEVKENILEESQVNDDIFNSLVKSVQQEQQHNVEEKVEESVEENDEESVEEN
VEENVEENDDESVASSVEESIASSVDESIDSSIEENVAPTVEEIVAPTVEEIVAPSVVESVAPSVEESVEENVEESVAENVEESVAENVEESVAENVEESVAENVEESVAENVEESVAENVE
EIVAPTVEESVAPTVEEIVAPSVEESVAPSVEEIVVPTVEESVAENVEEIVAPSVEEIVAPSVEEIVAPTVEESVAPTVEEIVAPSVEESVAPSVEEIVVPTVEESVAENVEESVAENVEEIVAPS
VEEIVAPSVEEIVAPSVEEIVAPSVEEIVAPSVEEIVAPSVEEIVAPSVEEIVAPSVEEIVAPTVEEIVAPTVEEIVAPSVEEIVAPTVEESVAENVATNLSDNLLSNLLGGIETEEIKDSILNEIEEV
KENVVTTILENVEETTAESVTTFSNILEEIQENTITNDTIEEKLEELHENVLSAALENTQSEEEKKEVIDVIEEVKEEVATTLIETVEQAEEESASTITEIFENLEENAVESNENVAENLEKLNETV
FNTVLDKVEETVEISGESLENNEMDKAFFSEIFDNVKGIQENLLTGMFRSIETSIVIQSEEKVDLNENVVSSILDNIENMKEGLLNKLENISSTEGVQETVTEHVEQNVYVDVDVPAMKDQ
FLGILNEAGGLKEMFFNLEDVFKSESDVITVEEIKDEPVQKEVEKETVSIIEEMEENIVDVLEEEKEDLTDKMIDAVEESIEISSDSKEETESIKDKEKDVSLVVEEVQDNDMDESVEKVLELK
NMEEELMKDAVEINDITSKLIEETQELNEVEADLIKDMEKLKELEKALSEDSKEIIDAKDDTLEKVIEEEHDITTTLDEVVELKDVEEDKIEKVSDLKDLEEDILKEVKEIKELESEILEDYKELKTI
ETDILEEKKEIEKDHFEKFEEEAEEIKDLEADILKEVSSLEVEEEKKLEEVHELKEEVEHIISGDAHIKGLEEDDLEEVDDLKGSILDMLKGDMELGDMDKESLEDVTAKLGERVESLKDVLSS
ALGMDEEQMKTRKKAQRPKLEEVLLKEEVKEEPKKKITKKKVRFDIKDKEPKDEIVEVEMKDEDIDEDIEEDVEEDIEEDKVEDIDEDIDEDIDEDIGEDKDEVIDLIVQKEKRIEKVKEKKK
KLEKKVEEGVSGLKKHVDEVMKYVQKIDKEVDKEVSKALESKNDVTNVLKQNQDFFSKVKNFVKKYKVFAAPFISAVAAFASYVVGFFTFSLFSSCVTIASSTYLLSKVDKTINKNKERPFY
SFVFDIFKNLKHYLQQMKEKFSKEKNNNVIEVTNKAEKKGNVQVTNKTEKTTKVDKNNKVPKKSRTQKSK

Figure S6

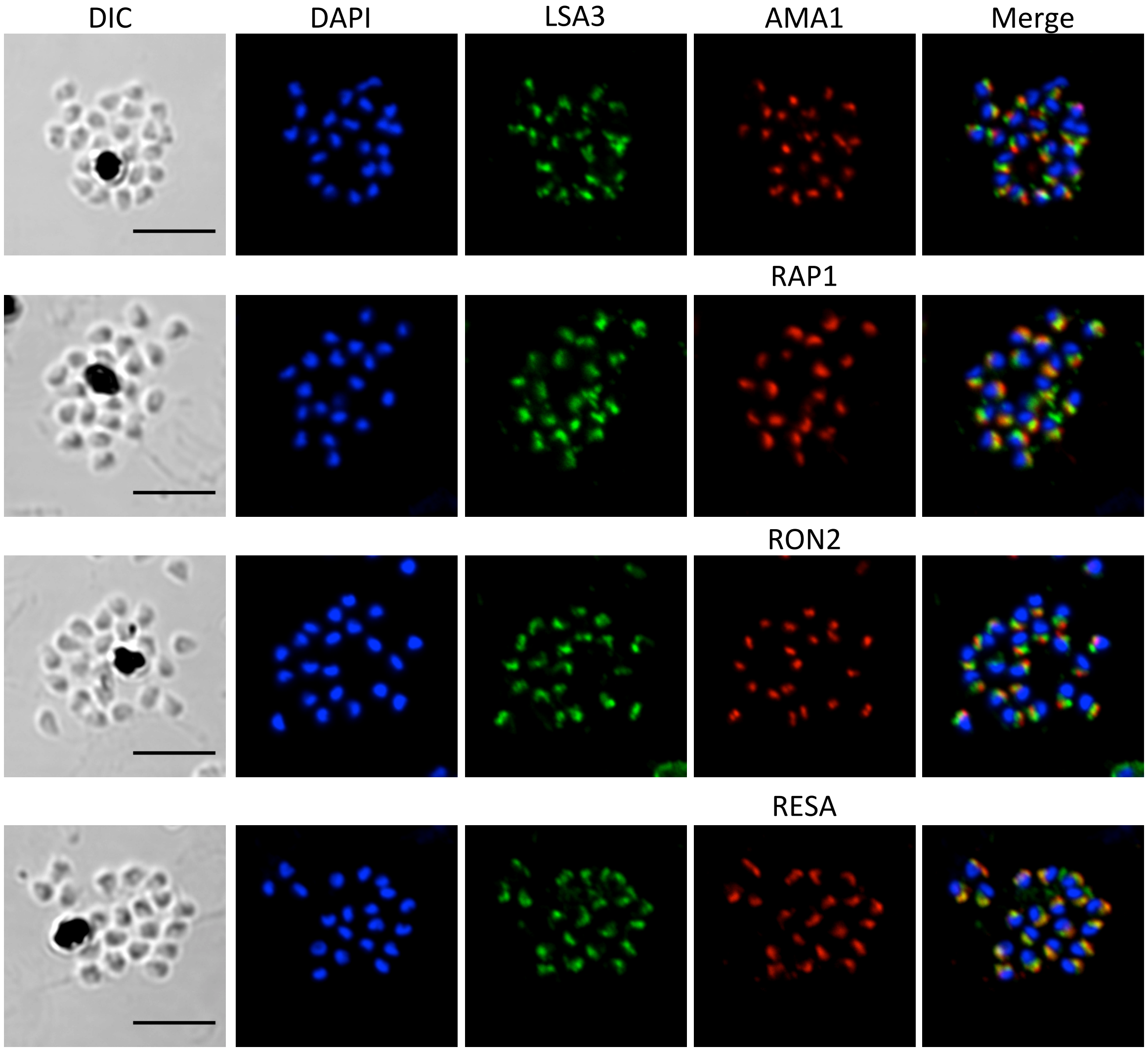

Figure S7

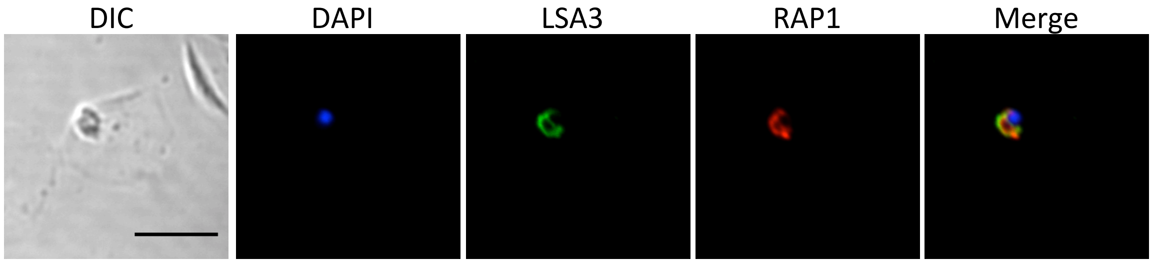

Supplement: Supplementary Information [file srep46086-s1.pdf]
